# Corrosion Resistance of Cr_2_O_3_, ZrO_2_, and Mn_3_O_4_ Oxide Films to Lead–Bismuth Eutectic: A Comparative Study

**DOI:** 10.3390/ma17235694

**Published:** 2024-11-21

**Authors:** Tao Liu, Wenguan Liu, Chongdou Yang, Penghui Lei, Di Yun, Jie Qiu

**Affiliations:** 1 School of Nuclear Science and Technology, Xi’an Jiaotong University, Xi’an 710049, China; 4123103234@stu.xjtu.edu.cn (T.L.); 425364@stu.xjtu.edu.cn (C.Y.); diyun1979@mail.xjtu.edu.cn (D.Y.); 2 Sino-French Institute of Nuclear Engineering and Technology, Sun Yat-sen University, Zhuhai 519082, China; liuwg7@mail.sysu.edu.cn

**Keywords:** lead–bismuth eutectic, oxide film, corrosion resistance, oxidation

## Abstract

In this study, the stability of Cr_2_O_3_, ZrO_2_, and Mn_3_O_4_ oxide films in high-temperature liquid lead–bismuth eutectic (LBE) was systematically investigated using both experimental and first principles calculation methods. The research findings indicated that Cr_2_O_3_ demonstrated superior structural integrity at corrosion temperatures of both 600 °C and 700 °C and displayed exceptional resistance to LBE corrosion. ZrO_2_ demonstrates resistance to LBE infiltration. However, the emergence of cracks in the vicinity of the ZrO_2_ layer and the metal interface undermines the protective layer’s integrity. Mn_3_O_4_ exhibits susceptibility to corrosion by LBE and lacks resistance to its effects. First principles calculations indicate that Pb and Bi atoms are most readily adsorbed onto the Mn_3_O_4_ surface, promoting the detachment of Mn atoms. The results show that the corrosion resistance of the three oxide films is ranked in the following order: Cr_2_O_3_ > ZrO_2_ > Mn_3_O_4_.

## 1. Introduction

Lead-cooled fast reactor (LFR) is a cutting-edge nuclear reactor technology that utilizes liquid lead or lead–bismuth eutectic (LBE) as a coolant, distinguishing it from conventional water-cooled reactors [1]. The LFR has several advantages, including a simplified structure, high power density, favorable neutron economy, enhanced thermal efficiency, and a reduction in long-lived nuclear waste [2]. However, the highly corrosive nature of liquid lead, or LBE, presents a significant challenge in terms of material resistance to corrosion [3,4]. Consequently, a thorough investigation of the corrosion behavior of structural materials is considered essential for advancing LFR.

An effective strategy to mitigate corrosion involves the promotion of a uniformly dense oxide layer formation on the material surface by regulating oxygen concentration in LBE [5,6,7]. To date, the oxidation behavior of martensitic/austenitic stainless steels in liquid LBE has been extensively investigated. In an oxygen-enriched LBE environment, the surface of stainless steel typically exhibits a stratified oxide structure, comprising an outer layer of magnetite (Fe_3_O_4_), a sublayer of compact spinel, and an innermost chromium-enriched oxide zone known as the internal oxidation zone (IOZ) [8,9,10]. The resistance to LBE corrosion primarily depends on the dense spinel inner oxide layer, whereas the porous Fe_3_O_4_ outer layer cannot provide enduring protective benefits. However, the iron-based oxide film is soluble in LBE at temperatures above 550 °C and thus cannot meet the high-temperature development requirements for LFRs [11,12,13]. The formation of oxide films is significantly influenced by alloying elements. It has been established that the addition of specific elements, such as aluminum (Al) and silicon (Si), to steel can promote the formation of a dense oxide layer, thereby enhancing its resistance to corrosion in LBE. For instance, Shen et al. [14] identified the formation of an Al_2_O_3_ layer on the surface as the principal contributor to the superior corrosion resistance of alumina-forming austenitic steels. Shi et al. [15] reported that the addition of Al led to the formation of an Al_2_O_3_ film, which enhanced the corrosion resistance of austenitic steels to LBE. Zhang et al. [16] demonstrated that SIMP steel containing 1.22 wt.% Si could form a continuous amorphous SiO_2_ film, which strengthens the adjacent Cr_2_O_3_ layer and improves oxidation resistance. Extensive research has been conducted on the effect of Al and Si on the corrosion resistance of alloys in LBE. However, research on alloying elements, such as chromium (Cr), zirconium (Zr), and manganese (Mn), is relatively scarce, but some experiments have shown that these elements can improve the oxidation properties of the oxide layer. Wang et al. [17] investigated the LBE corrosion resistance of four Fe–Cr–Al alloys with varying Cr concentrations and observed an inverse relationship between Cr content and the thickness of the oxide layer formed after corrosion. Jin et al. [18] suggested that the formation of manganese oxide reduces the oxygen partial pressure within the corrosion layer, thereby facilitating the external oxidation of Cr, and enhancing the alloy’s resistance to high-temperature oxidation. Gao [19] and Takaya et al. [20] demonstrated that the addition of a small amount of Zr can prevent the coarsening of oxide particles in oxide dispersion-strengthened (ODS) steels, which is induced by Al, thereby improving the performance of ODS steels. To understand the corrosion behavior and rank different alloy oxides for their suitability in liquid metal systems, systematic studies on the corrosion behavior of Cr, Mn, and Zr oxides in LBE are highly desirable, which are imperative for the development of structural materials resistant to high-temperature liquid LBE corrosion.

In this study, three metallic materials, namely, Cr, Zr, and Mn, were selected for investigation. These materials were pre-oxidized in air to form an oxide layer on their surfaces, followed by exposure to LBE at temperatures of 600 °C or 700 °C for a duration of 50 h. The corrosion experiments were conducted under an oxygen concentration range of 10^−6^~10^−5^ wt.%. The corrosion resistance of the three oxides was characterized and compared. First principles calculations were employed to calculate the adsorption energy of Pb and Bi atoms and the escape energy of alloy atoms on their surfaces and provide a basis for understanding their differential performance under LBE corrosive environments.

## 2. Experiment and Theoretical Calculation

### 2.1. Materials

**Figure 1:**
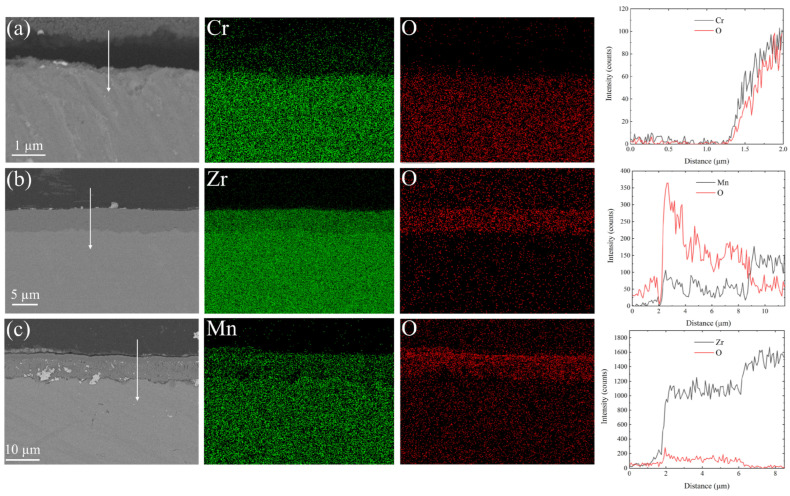
SEM micrographs and EDS mapping of (**a**) Cr, (**b**) Zr, and (**c**) Mn metal surfaces after pre-oxidation in air at 600 °C for 1 h, along with the corresponding line scan data obtained in the direction of the white arrows.

Three flake metals with Cr contents of 99.5%, Zr-4 alloy (Sn 1.2~1.7 wt%, Fe 0.18~0.24 wt.%, Cr 0.07~0.13 wt.%, O ∼ 0.16 wt.%, and Zr bal.), and Mn contents of 99.9% were chosen as experimental materials. The samples were ground mechanically with SiC sandpaper with grit sizes from 600 to 3000 and further polished with 0.5 μm alumina paste to achieve a mirror-like surface. The samples were then placed in a tube furnace and subjected to thermal treatment at 600 °C for 1 h in air, and finally, the oxidized samples were obtained. The scanning electron microscopy (SEM) micrographs of the three pre-oxidized samples are shown in Figure 1. The surfaces of Zr and Mn exhibited oxide layers with thicknesses of 4.69 µm and 6.71 µm, respectively. Despite the high magnification used, no distinct oxidation features were detected on the Cr surface.

**Figure 2:**
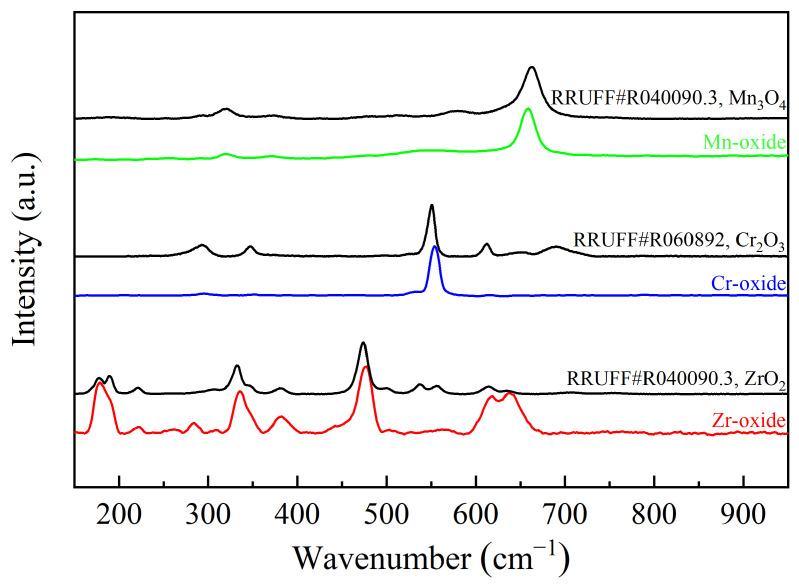
Raman spectra of the surface oxides of Cr, Zr, and Mn metals compared with the standard spectra of Cr_2_O_3_, ZrO_2_, and Mn_3_O_4_, respectively.

To further ascertain the composition of the surface oxides, Raman spectroscopy was conducted. The Raman spectra obtained were compared with the standard spectra from the RRUFF Database, and the results are presented in Figure 2. Following heat treatment, the Raman spectral data for manganese oxide corresponded to the wavenumber of the Mn_3_O_4_ standard spectrum, confirming the presence of Mn_3_O_4_ in the analyzed areas. Additionally, the Raman spectra of Cr and Zr shifted to higher wavenumbers compared to the standard spectra, indicating incomplete oxidation, as evidenced by the presence of Cr or Zr in the analyzed regions. The results indicate that Cr_2_O_3_, ZrO_2_, and Mn_3_O_4_ have formed on the surfaces of the Cr, Zr, and Mn metals, respectively.

### 2.2. Corrosion Tests

The oxidized samples were immersed in LBE, which was composed of 55.5 wt.% bismuth and 44.5 wt.% lead, and placed in a box muffle furnace under oxygen concentrations ranging from 10^−6^ to 10^−5^ wt.%. They were subjected to corrosion at temperatures of 600 °C and 700 °C for 50 h, respectively. After the corrosion tests, the samples were removed from the LBE and embedded in resin without cleaning the LBE for subsequent cross-sectional examination. The cross-sectional samples were polished with SiC papers graded from 400 to 3000 grit and then mechanically polished with a 0.5 μm alumina polish to achieve a mirror-like finish. The samples were analyzed using a scanning electron microscope (SEM, Gemini 500, ZEISS, Oberkochen, Germany) equipped with an energy-dispersive spectrometer (EDS).

### 2.3. First Principles Method and Computational Models

In the framework of density-functional theory (DFT) [21,22], the VASP (6.4.0) software [23] was utilized to conduct spin-polarized electronic state calculations. The interaction between electrons and ions was characterized using the projector augmented wave (PAW) [24] method, with the wave function expanded on a plane–wave basis set to a cutoff energy of 500 eV. The exchange correlation energy was computed within the generalized gradient approximation (GGA) using the Perdew–Burke–Ernzerhof (PBE) method [25]. Convergence criteria were used for the total energy and Hellmann–Feynman forces to achieve convergence at 10^−5^ eV and 0.02 eV/Å, respectively.

The Cr_2_O_3_ crystal model, which was retrieved from the Crystallography Open Database (COD) with the identifier COD #9016728, features a hexagonal close-packed (HCP) structure. The Cr_2_O_3_ crystals demonstrate an antiferromagnetic arrangement, with chromium atoms along the [001] direction exhibiting alternating magnetic moments that switch between upward and downward orientations. The lattice utilized a 7 × 7 × 3 Monkhorst–Pack k-point grid for sampling the Brillouin zone. The initial magnetic moment of the Cr atoms is 2.67 $\mu_{B}$. The optimized lattice parameters are a = b = 4.944 Å and c = 13.800 Å, which are in good agreement with previously reported data in the literature [26,27]. For the (0001) plane, the configuration with the outermost layer consisting of Cr atoms and the second layer of O atoms is identified as the most stable [26]. Therefore, this atomic arrangement of the (0001) plane is selected for computational analysis. The dimensions of the Cr_2_O_3_ surface model were 9.89 × 9.89 × 26.36 Å^3^. During the calculations, the k-points were set to a grid of 3 × 3 × 1.

ZrO_2_ exhibits three stable phases across different temperature ranges. The monoclinic phase (P21/c) is stable under ambient conditions (0 to 1180 °C) [28], while the tetragonal phase (P42/nmc) is stable between 1180 to 2370 °C, and the cubic phase (Fm-3m) is stable at even higher temperatures, ranging from 2370 to 2600 °C [29,30,31]. In this work, the monoclinic phase structure of ZrO_2_ was utilized. For the optimization of the ZrO_2_ unit cell, a k-point mesh of 7 × 7 × 7 was employed. The optimized lattice parameters were determined to be a = 5.181 Å, b = 5.255 Å, and c = 5.355 Å, which is consistent with the data in the literature [32,33]. Monoclinic zirconia exhibits nine distinct crystallographic orientations: [001], [010], [100], [110], [101], [011], [$\bar{1}$01], [111], and [$\bar{1}$11] [30]. A theoretical analysis indicates that the ($\bar{1}$11) surface is the most stable among these orientations for monoclinic zirconia [30]. Consequently, the ZrO_2_($\bar{1}$11) surface was modeled for theoretical calculations, with the dimensions set to 6.81 × 7.38 × 30.37 Å^3^ and the k-points mesh adjusted to 5 × 5 × 1.

Paulo et al. [34] provided a comprehensive discussion of the computational model of the antiferromagnetic oxide Mn_3_O_4_. They assessed the Mn_3_O_4_ cell using various kinetic cutoff energy, k-point grid, and spin magnetization values. Their findings indicated that the Mn_3_O_4_(001) surface is the most stable and serves as an appropriate model. Additionally, they conducted spin polarization tests, revealing that the most stable configuration was the spin-compensated system, which was characterized by a net magnetization of zero. The unit cell model of Mn_3_O_4_ was subsequently developed based on the one reported by Paulo [34]. The k-point mesh was configured to 7 × 7 × 5, and the optimized cell parameters were consistent with the data in the literature [34,35]. In this study, the spin polarization of Mn^2+^ and Mn^3+^ centers in the Mn_3_O_4_ cell were determined to be 4.2 and 3.6 $\mu_{B}$/atom, respectively, which aligns with the calculated results reported by Paulo [34]. For the Mn_3_O_4_(001) surface, the outermost layer, which was composed of Mn and O atoms, was identified as the most stable configuration [34]. The dimensions of the Mn_3_O_4_ surface model are 11.55 × 11.55 × 25.94 Å^3^, with a k-point mesh set to 3 × 3 × 1.

**Figure 3:**
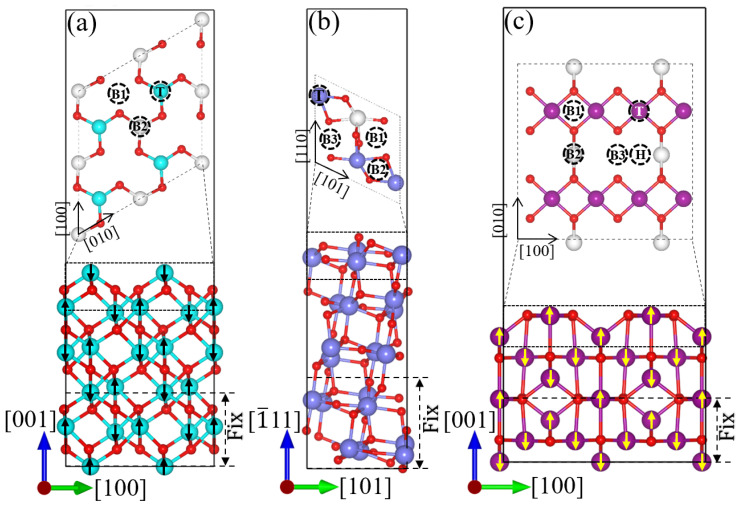
Modeling of three oxide layer structures is presented as follows: (**a**) Cr_2_O_3_(001), (**b**) ZrO_2_($\bar{1}$11), and (**c**) Mn_3_O_4_(001). The initial magnetic moments of the atoms are indicated by arrows, and the number of fixed atomic layers is labeled accordingly. The adsorption sites on the surface atomic layer are categorized into three types: bridge, hollow, and top sites. The initial letter of each site’s descriptor denotes the type of site, and the numerical suffix represents the quantity of such sites. White atoms represent inner atoms, which are used to differentiate between various adsorption site environments.

The surfaces were modeled with vacuum layers exceeding 15 Å to mitigate interactions arising from periodic boundary conditions. Figure 3a–c depict the modeled surface structures of Cr_2_O_3_(001), ZrO_2_($\bar{1}$11), and Mn_3_O_4_(001), respectively, along with the high-symmetry adsorption sites on each surface. The number of fixed atomic layers and the magnitudes of positive and negative magnetic moments are indicated in Figure 3. Three types of adsorption sites are identified: bridge, hollow, and top sites. The initial letter of each site’s name specifies the type, and the numerical suffix denotes the count of such sites. The initial magnetic moment orientations for the antiferromagnetic surface models of Cr_2_O_3_ and Mn_3_O_4_ are represented by arrows. A comparison of the optimized cell parameters presented in this work with both computational and experimental data is provided in Table 1.

## 3. Results

### 3.1. Corrosion Experiment Results

#### 3.1.1. Corrosion Experiment at 600 °C

**Figure 4:**
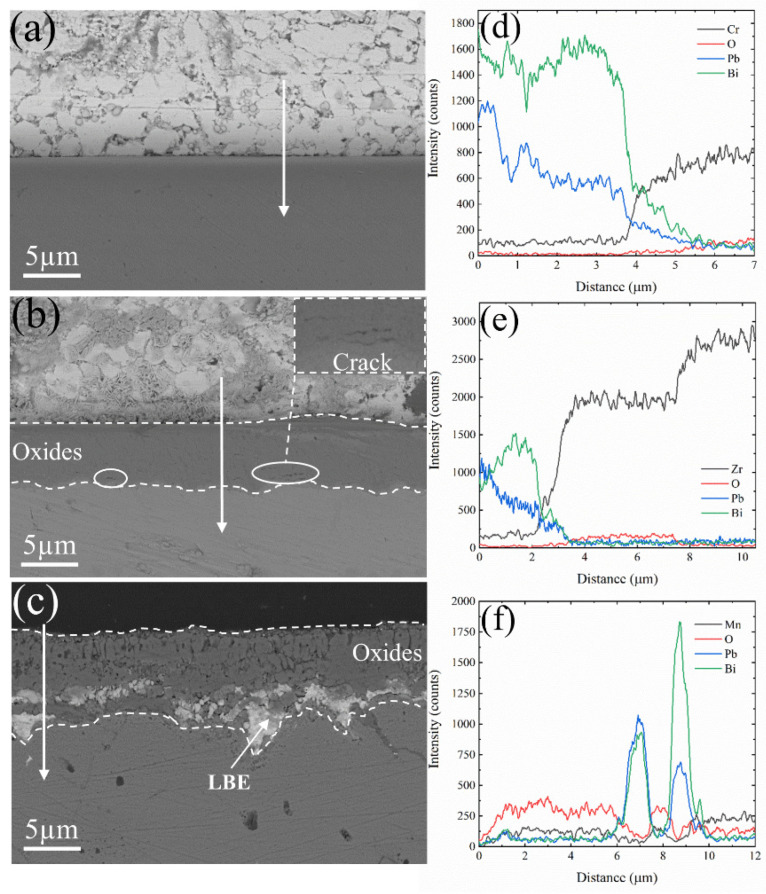
Cross-sectional morphology of oxidized (**a**) Cr, (**b**) Zr, and (**c**) Mn after corrosion in liquid LBE at 600 °C for 50 h; (**d**), (**e**), and (**f**) correspond to the line scan data taken along the direction of the white arrows in (**a**), (**b**), and (**c**), respectively.

**Figure 5:**
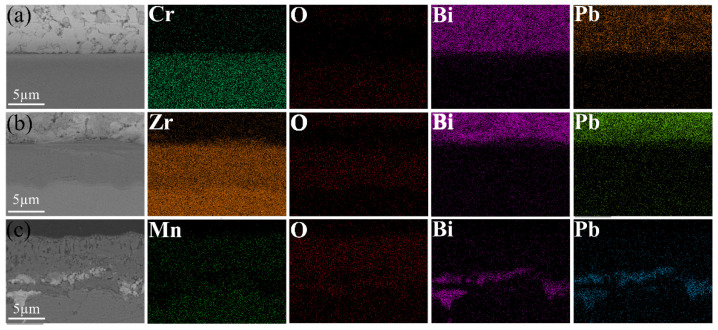
EDS mapping of oxidized (**a**) Cr, (**b**) Zr, and (**c**) Mn exposed to liquid LBE at 600 °C for 50 h.

Figure 4 illustrates the cross-sectional morphologies and corresponding line scan data of Cr, Zr, and Mn oxides after 50 h of corrosion in liquid LBE at 600 °C. The oxide film on the surface of Cr metal was exceedingly thin, rendering it challenging to resolve with SEM. In contrast, Zr and Mn metal surfaces developed oxide films with thicknesses of 5.8 µm for ZrO_2_ and 7.6 µm for Mn_3_O_4_. Oxidation studies on pure chromium metal have confirmed the excellent compactness of Cr_2_O_3_. Yang et al. [36] reported that exposing metallic Cr to air at 500 °C for 2 h resulted in the formation of an oxide film approximately 100 nm thick on the surface. Hallström et al. [37] conducted a comparable oxidation experiment, revealing that the thickness of the Cr_2_O_3_ oxide film formed on pure Cr surfaces ranged from 290 to 430 nm after being exposed to dry oxygen at 625 °C for 24 h. The Cr_2_O_3_ generated in the initial stages effectively isolates oxygen, thereby preventing the further oxidation of chromium and inhibiting the increase in oxide layer thickness. The EDS results depicted in Figure 5 demonstrate that both Cr_2_O_3_ and ZrO_2_ exhibit excellent resistance to lead–bismuth corrosion. In contrast, Mn_3_O_4_ shows a significant number of voids and cracks within its structure, accompanied by evident lead–bismuth infiltration, indicating that Mn_3_O_4_ lacks resistance to high-temperature liquid LBE corrosion. To enhance the adhesion of ZrO_2_ coatings, Zhu et al. [38,39] introduced a Cr interlayer between the coating and the substrate to alleviate the stress induced by the different thermal expansion coefficients at elevated temperatures. Despite being covered by ZrO_2_, the Cr layer remains susceptible to oxidation, indicating that ZrO_2_ cannot prevent the permeation of oxygen. Figure 4 illustrates the presence of minor cracks in the ZrO_2_ oxide layer near the substrate, which may be attributed to stress concentration at the ZrO_2_–substrate interface.

#### 3.1.2. Corrosion Experiment at 700 °C

**Figure 6:**
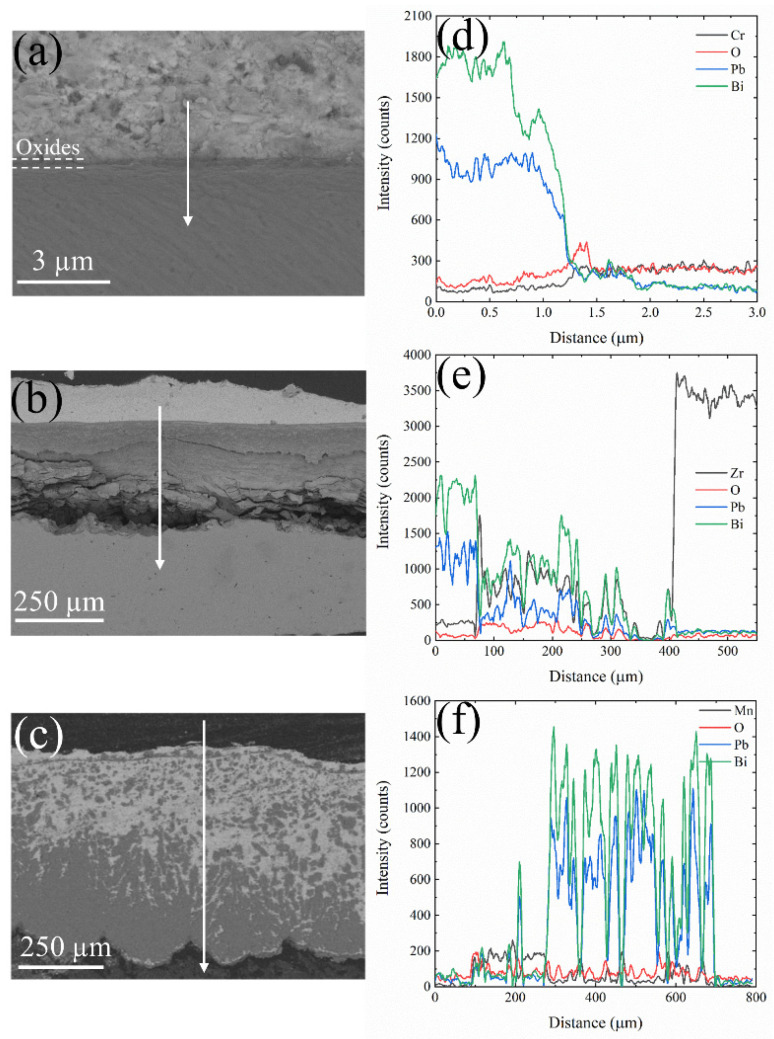
Cross-sectional morphology of oxidized (**a**) Cr, (**b**) Zr, and (**c**) Mn after corrosion in liquid LBE at 700 °C for 50 h; (**d**), (**e**), and (**f**) correspond to the line scan data taken along the direction of the white arrow in (**a**), (**b**), and (**c**), respectively.

**Figure 7:**
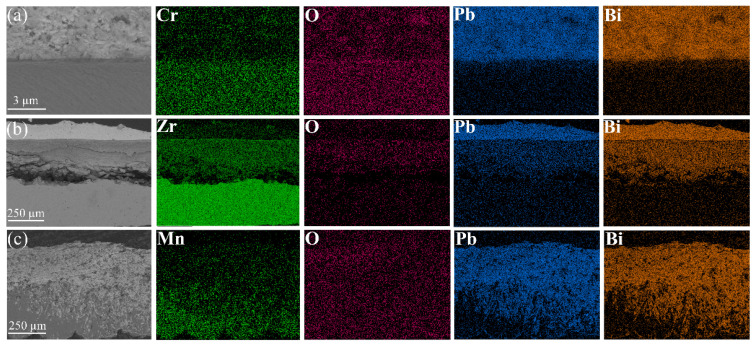
EDS mapping of oxidized (**a**) Cr, (**b**) Zr, and (**c**) Mn after corrosion in liquid LBE at 700 °C for 50 h.

Cr_2_O_3_ and ZrO_2_ demonstrate effective resistance to lead–bismuth corrosion during short-term exposure at 600 °C. To further verify the resistance of the oxide film in LBE at elevated temperatures, a corrosion experiment was conducted at 700 °C for 50 h, and the corrosion morphology is shown in Figure 6. A distinct oxide film forms on the surface of Cr metal, with line scan results indicating a significant increase in O elemental counts at the location of the oxide layer. Even at 700 °C in liquid LBE, Cr_2_O_3_ maintains its excellent resistance to corrosion. However, the considerable thickening of the oxide film on the Zr metal surface and the formation of internal cracks may lead to the detachment of the ZrO_2_ oxide film. The line scan results presented in Figure 6b reveal the presence of Pb and Bi within the oxide layer. Additionally, the EDS analysis shown in Figure 7b demonstrates the penetration of Pb and Bi into the oxide film, indicating a loss of its protective properties. At 700 °C in liquid LBE, the oxide film on the Mn metal surface was dissolved by LBE, leading to nearly complete corrosion of the substrate, as illustrated in Figure 7c. The line scan data further indicate that the Mn metal is saturated with LBE, and it has been demonstrated that Mn_3_O_4_ is vulnerable to high-temperature LBE.

### 3.2. First Principles Calculations

#### 3.2.1. Corrosion Resistance of Oxide Layers

The corrosion behavior of oxides in LBE can be characterized through adsorption and escape energies [40,41]. Adsorption energy reflects the stability of the system in its final adsorbed state, and the escape energy quantifies the influence of adsorbed atoms on the surface alloying elements. The adsorption stability of an atom on the surface is defined by the adsorption energy, which can be calculated using the following equation:

(1) $$E_{ads}^{i}=E_{surface}^{ads-i}-E_{surface}-\mu_{i}$$

where $E_{surface}^{ads-i}$ and $E_{surface}$ are the total energies of the slab with and without an adatom, respectively, and $\mu_{i}$ is the chemical potential of the adatom (i.e., Pb or Bi atom). Ignoring the contributions of free energy and latent heat of fusion with respect to temperature dependence, the value of $\mu_{X}$ at 0 K is consistent with the energy per atom of the most stable condensed phase of the element, regardless of the type of adatom [42]. A positive adsorption energy indicates that the system is energetically unfavorable and unstable in the adsorption state. Conversely, a negative $E_{ads}^{X}$ description system has a more favorable energy advantage.

The strength of the binding of the surface structure to atoms can be characterized by the escape energy, which is defined as follows:

(2) $$E_{esp}^{i}=E_{surface-X}^{ads-i}+E_{X}-E_{surface}^{ads-i}$$

where $E_{surface-X}^{ads-i}$ represents the total energy associated with the presence of an adatom on a surface from which an alloy atom (i.e., Cr, Zr or Mn) has detached. *i* = 0 means there are no adatom on the surface. $E_{X}$ is the energy of an isolated alloy atom. A lower escape energy indicates that an atom is more likely to detach from the structure. The adsorption and escape energies can be utilized to accurately estimate the capacity of liquid LBE to dissolve and corrode the oxide layer.

**Figure 8:**
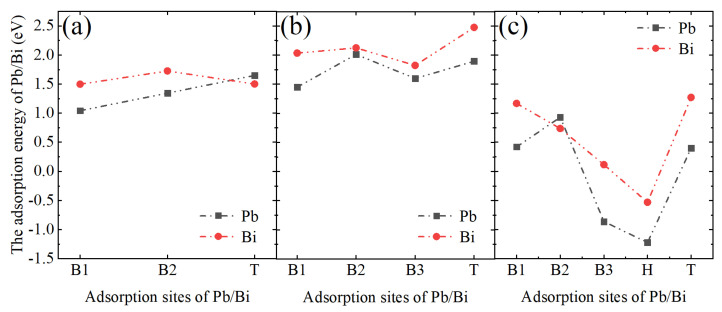
Adsorption energies of Pb and Bi atoms at each high-symmetry site on (**a**) Cr_2_O_3_, (**b**) ZrO_2_, and (**c**) Mn_3_O_4_ oxide layers. See Figure 3 to identify the adsorption site.

Figure 8a–c show the adsorption energies of Pb and Bi atoms at each high-symmetry site of the oxidized layers of Cr_2_O_3_, ZrO_2_, and Mn_3_O_4_, respectively. The adsorption energies of Pb and Bi were found to be positive at all sites on the Cr_2_O_3_ and ZrO_2_ surfaces. Moreover, the adsorption energies on the Mn_3_O_4_ surface were generally lower than those on the others. In addition, the adsorption energies of Pb and Bi on the B3 and H sites of Mn_3_O_4_ were negative, indicating that Pb and Bi are easily adsorbed on the Mn_3_O_4_ surface. It is noteworthy that the adsorption energies of Pb atoms on the three surfaces are generally lower than those of Bi. Previous corrosion calculations on Fe_3_O_4_ surfaces have reached similar conclusions [41], suggesting that Pb is more readily adsorbed on the oxide layers than Bi.

**Figure 9:**
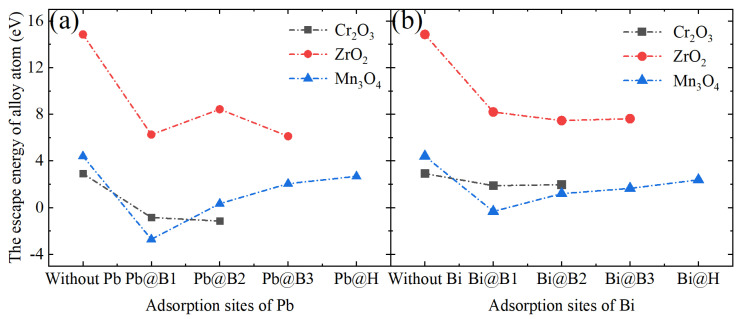
Escape energy of the outermost alloying elements on the three surfaces and the escape energy of the alloying elements in proximity to the stable adsorption site of (**a**) Pb or (**b**) Bi atoms.

The escape energy quantifies the ease with which an alloying element can detach from an oxidized surface. In the absence of other adsorbed atoms, the escape energies of the alloying elements on the surfaces of Cr_2_O_3_, ZrO_2_, and Mn_3_O_4_ were measured to be 2.92 eV, 14.84 eV, and 4.41 eV, respectively. This indicates that the Zr atoms on the ZrO_2_ surface are the least likely to escape, followed by Mn and Cr atoms. There is a significant difference in the escape energy of alloying elements between ZrO_2_ and the other oxides, which may be associated with the structure of the oxide layer and the oxygen coordination number of these elements. The Zr atoms on the monoclinic phase ZrO_2_($\bar{1}$11) exhibit the highest oxygen coordination. Figure 9a,b depict the escape energy of neighboring alloying elements in the adsorbed states of Pb and Bi atoms, respectively. The results show that the adsorption of Pb and Bi on the surface results in a reduction of the escape energy for the alloying elements. By considering the adsorption energies depicted in Figure 8 and the escape energy data presented in Figure 9, it can be inferred that Mn is most likely to detach from the Mn_3_O_4_ surface in the presence of adsorbed Pb and Bi atoms.

#### 3.2.2. Ab Initio Molecular Dynamics Calculations of the Solid–Liquid Interface

**Figure 10:**
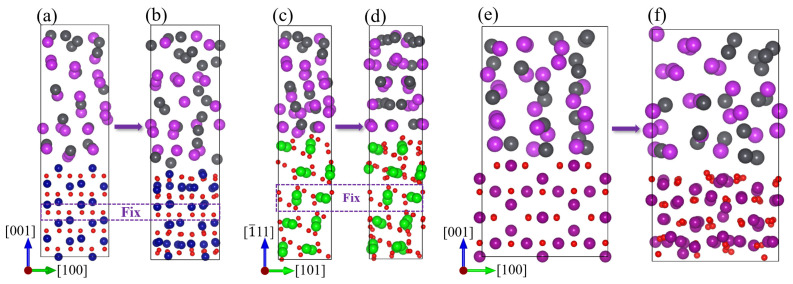
(**a**) and (**b**) show the solid–liquid interface model of the Cr_2_O_3_(001) surface with liquid LBE at 0 and 10,000 steps of optimization, respectively. (**c**,**d**) represent the ZrO_2_($\bar{1}$11) surface with liquid LBE. (**e**,**f**) illustrate the Mn_3_O_4_(001) surface with liquid LBE. The purple-dotted boxes represent the fixed atomic layers.

To investigate the interfacial interactions among Cr_2_O_3_(001), ZrO_2_($\bar{1}$11), and Mn_3_O_4_(001) surfaces and liquid LBE, as well as their stability differences, ab initio molecular dynamics (AIMD) simulations were conducted. The temperature was set to 600 °C to align with experimental conditions. AIMD simulations were performed for 10,000 steps in the NVT ensemble for each system, each with a time step of 1 fs. The interfaces of Cr_2_O_3_(001), ZrO_2_($\bar{1}$11), and Mn_3_O_4_(001) surfaces with liquid LBE were modeled as shown in Figure 10a,c,e. The solid–liquid interface model of Cr_2_O_3_ (001) surface and liquid LBE is 9.89 × 9.89 × 29.23 Å^3^, with 40 Cr, 60 O, 19 Pb, and 23 Bi atoms, and the liquid LBE has a thickness of 14.96 Å. The solid–liquid interface model of ZrO_2_($\bar{1}$11) surface and liquid LBE is 13.62 × 7.38 × 29.02 Å^3^, with 40 Zr, 80 O, 19 Pb, and 23 Bi atoms, and the liquid LBE has a thickness of 13.75 Å. The solid–liquid interface model of the Mn_3_O_4_(001) surface and liquid LBE is 11.55 × 11.55 × 21.15 Å^3^, with 48 Mn, 64 O, 19 Pb, and 23 Bi atoms, and the liquid LBE has a thickness of 10.35 Å. The vacuum layer is not retained in order to preserve the LBE and maintain the liquid density, so there are two interfaces. The density of the liquid LBE is 10.5 g/cm^3^. Figure 10b,d,f show the solid–liquid interfacial models of Cr_2_O_3_ (001), ZrO_2_($\bar{1}$11), and Mn_3_O_4_(001) surfaces with liquid LBE after 10,000 steps of relaxation, respectively. The disorder of atomic arrangement on the Mn_3_O_4_(001) surface is the most serious after relaxation, whereas the Cr_2_O_3_(001) and ZrO_2_($\bar{1}$11) surfaces predominantly maintain their initial structures. This suggests that the Mn_3_O_4_(001) surface is particularly susceptible to the perturbing effects of Pb and Bi, which can lead to structural degradation.

## 4. Discussion

Three pure metals of Cr, Zr, and Mn were pre-oxidized in air at 600 °C. Raman spectroscopy analysis confirmed the formation of Cr_2_O_3_, ZrO_2_, and Mn_3_O_4_ oxide films on their respective surfaces. Subsequently, high-temperature LBE corrosion experiments were conducted in an environment with an oxygen concentration ranging from 10^−6^ to 10^−5^ wt.%. As illustrated in Figure 4, the Cr_2_O_3_ oxide layer is notably thin, and the thickness of the ZrO_2_ and Mn_3_O_4_ oxide films are 5.8 µm and 7.6 µm, respectively. Yang et al. [36] observed the formation of a thin Cr_2_O_3_ oxide layer during the oxidation of Cr in air, which was characterized by a gradient distribution of elemental content. The oxygen content was found to decrease from the outermost layer toward the inner layer, while the chromium content exhibited a gradient in the opposite direction, suggesting an element diffusion process. According to the research results of Lim [43] and Ma [44] et al., the diffusion coefficient of oxygen in Cr_2_O_3_ and ZrO_2_ can be obtained using Equations (3) and (4), as follows:

(3) $$D_{O-{Cr}_{2}O_{3}}=15.9\times exp(\frac{-421,747(J/mol)}{RT}){cm}^{2}/s$$

(4) $$D_{O-ZrO_{2}}=0.115\times exp\left(\frac{-143,640\left(J/mol\right)}{RT}\right){cm}^{2}/s$$

The diffusion coefficients of oxygen in Cr_2_O_3_ and ZrO_2_ are 9.24 × 10^−25^ cm^2^/s and 2.92 × 10^−10^ cm^2^/s at 600 °C, respectively. The results indicate that the diffusion rate of oxygen in Cr_2_O_3_ is much lower than that in ZrO_2_, and Cr_2_O_3_ can effectively hinder the diffusion of oxygen. Therefore, the formed Cr_2_O_3_ oxide layer remains very thin.

As depicted in Figure 4 and Figure 6, Cr_2_O_3_ demonstrates superior resistance to LBE corrosion at elevated temperatures of 600 °C and 700 °C. The Cr_2_O_3_ layer is characterized by its high density and slow growth rate, which alleviates the stress issues that are typically associated with excessively thick oxide layers. Wang et al. [45] coated a martensitic steel surface with a layer of metallic chromium, and the resultant Cr_2_O_3_ oxide layer significantly enhanced the corrosion resistance of the martensitic steel. As shown in Figure 6, the thickness of Cr_2_O_3_ increases with increasing temperature, with the oxide layer thickness being approximately 160 nm at 700 °C. The adsorption energy results depicted in Figure 8 indicate that Pb and Bi atoms achieve larger adsorption energies on the Cr_2_O_3_ surface, preferentially adsorbing at the B1 site, which is coordinated by three oxygen atoms and unaffected by interference from inner Cr atoms. Figure 9 illustrates that among the three oxide layers, the pristine Cr_2_O_3_ has the lowest escape energy for Cr atoms. However, with the adsorption of Pb and Bi, the reduction in escape energy for Cr atoms is minimal, implying the least susceptibility to the effects of Pb and Bi. The AIMD simulation results presented in Figure 10 show that Cr_2_O_3_ maintains a relatively intact crystal structure after prolonged relaxation, reflecting its structural stability. The computational results suggest that Cr_2_O_3_ possesses excellent resistance in LBE, which is consistent with experimental findings.

ZrO_2_ has demonstrated excellent resistance to LBE corrosion at 600 °C. Zhu et al. [38,39] conducted comprehensive studies on the LBE corrosion resistance of ZrO_2_ coatings, and their findings indicate that ZrO_2_ exhibits superior resistance to LBE corrosion. However, the EDS results shown in Figure 4 reveal the presence of minor cracks near the substrate, which could potentially reduce the corrosion resistance of ZrO_2_. Kurpaska [46] and Jordan et al. [47] have reported that oxidizing zirconium metal in air or oxygen at 600 °C for an extended period led to the formation of similar cracks in the oxide layer. This phenomenon may be attributed to the pronounced stress relaxation gradient within the ZrO_2_ oxide film in that region, resulting in the formation of relatively large cracks [46]. In corrosion experiments conducted at 700 °C, the thickness of the ZrO_2_ oxide layer increased from 5.8 µm at 600 °C to 310 µm, as depicted in Figure 6b. According to Equation (4), the diffusion rate of oxygen in ZrO_2_ at 700 °C is calculated to be 2.23 × 10^−9^ cm^2^/s, which significantly accelerates the oxidation process of Zr metal. Alansari et al. [48] investigated the impact of oxidation time on the Zr metal oxide layer at 650 °C. Their findings indicate that a dense, non-porous, and adherent zirconium dioxide layer can be formed on the surface when the oxidation time is less than 12 h. However, with an extended oxidation time, pores gradually develop within the oxide layer. The detachment of the oxide layer is characterized by accelerated oxidation kinetics and the formation of cracks, which may be associated with stress changes in ZrO_2_ at elevated temperatures. Kurpaska et al. [49] conducted measurements of growth stress during the high-temperature oxidation of pure zirconium over a period of 24 h. They observed that the compressive stress increased almost continuously, reaching 2.8 Gpa at 400 °C. Upon raising the temperature to 500 °C, the compressive stress increased to 5.5 Gpa within the initial hours of the experiment, followed by stress relaxation. Overall, the compressive stress increases with temperature during oxidation, and stress relaxation occurs due to oxide cracking near the metal/oxide interface after reaching a critical value [50,51]. This phenomenon explains the oxide cracking observed in Figure 4b and Figure 6b. As illustrated in Figure 7, under the influence of Pb and Bi, ZrO_2_ maintains the highest escape energy, indicating that Zr does not readily detach from the oxide surface. ZrO_2_, despite its promising theoretical data, can develop stress-induced cracking due to the growth of the oxide layer, which leads to the generation of cracks and diminishes its resistance to lead–bismuth corrosion in experimental conditions, compared to what theoretical calculations might suggest. It should be noted that in the calculation process of first principles, the system temperature is usually performed at 0 K, and experimental temperature conditions cannot be considered, representing a limitation of theoretical calculations. Additionally, the results from AIMD simulations indicate that the ZrO_2_ structure demonstrates remarkable stability. In fact, ZrO_2_ exhibits significant resistance to LBE corrosion. However, the existing oxidation issue leads to the formation of cracks, which compromise the integrity of the ZrO_2_ layer. This suggests that the zirconium oxide layer is susceptible to failure due to oxidation at elevated temperatures.

In corrosion experiments conducted at 600 °C, Mn_3_O_4_ was corroded and penetrated by LBE, resulting in completely losing its protective function, as shown in Figure 4c. When the temperature was increased to 700 °C, Mn_3_O_4_ was dissolved, and the substrate was inundated with a significant amount of LBE, as depicted in Figure 6c. This indicates that Mn_3_O_4_ lacks the ability to resist LBE corrosion. Wang et al. [52], in their study of the corrosion of high manganese alloys, reported that the corrosion resistance of the Mn oxides formed on the material surface was inadequate, and LBE penetration into the interior of the oxide layer was observed. As shown in Figure 8, the adsorption energies of Pb and Bi on the surface of Mn_3_O_4_ are generally lower than those on the other surfaces, indicating a higher tendency for Pb and Bi to adsorb on the Mn_3_O_4_ surface. Furthermore, Mn_3_O_4_ exhibits the lowest escape energy for Mn atoms, suggesting that it is susceptible to the influence of Pb and Bi, which lead to detachment from the surface. The AIMD simulations depicted in Figure 10 reveal that after relaxation, the atomic arrangement on the surface of Mn_3_O_4_ is the most severely disordered, resulting in significant compromise of its surface structure. This further corroborates the conclusion that Mn_3_O_4_ faces considerable challenges in resisting LBE corrosion.

## 5. Conclusions

In this study, the corrosion behavior of Cr_2_O_3_, ZrO_2_, and Mn_3_O_4_ oxides in liquid LBE at temperatures of 600 °C and 700 °C was investigated to explore their corrosion resistance in LBE. The following conclusions were drawn:

(1) At 600 °C, Cr, Zr, and Mn metals oxidize in air, forming Cr_2_O_3_, ZrO_2_, and Mn_3_O_4_ oxide layers on their respective surfaces. Among these oxides, Cr_2_O_3_ exhibits the lowest diffusion coefficient for oxygen, which hinders the oxidation of chromium through the oxide layer, resulting in the formation of the thinnest Cr_2_O_3_ layer. Oxygen atoms are more likely to continue reacting with the metal through ZrO_2_ and Mn_3_O_4_, leading to a further increase in the oxide layer thickness.
(2) Cr_2_O_3_ and ZrO_2_ exhibited excellent resistance to lead–bismuth corrosion at 600 °C. However, when the corrosion temperature was increased to 700 °C, ZrO_2_ developed a large number of cracks, resulting in a loss of its protective capability. In contrast, Mn_3_O_4_ could not withstand LBE corrosion. At 600 °C, a substantial amount of LBE penetrated Mn_3_O_4_, and after further increasing the corrosion temperature to 700 °C, Mn_3_O_4_ was completely corroded. The results show that the corrosion resistance of the three oxide films can be ranked in the following order: Cr_2_O_3_ > ZrO_2_ > Mn_3_O_4_.
(3) First principles calculations indicate that Pb and Bi atoms are most readily adsorbed onto the Mn_3_O_4_ surface. Under the influence of Pb or Bi atoms, Mn atoms are more likely to detach from the surface. Zr atoms on the ZrO_2_ surface possess a high escape energy, suggesting that ZrO_2_ theoretically exhibits excellent resistance to LBE corrosion. ZrO_2_ possesses excellent theoretical data; however, stress issues arising from the growth of the oxide layer result in the formation of cracks. At 600 °C, fine cracks within ZrO_2_ can be observed, but the overall integrity of the oxide film remains intact, thus it exhibits good resistance to lead–bismuth corrosion. Nevertheless, the proliferation of cracks becomes particularly pronounced at elevated temperatures, suggesting that ZrO_2_ does not perform as well as suggested by theoretical calculations. Provided that the integrity of the oxide film is maintained, ZrO_2_ holds significant application potential.

## Figures and Tables

**Table 1:** Comparison of the optimized cell parameters obtained in this study with both computational and experimental data from the literature.

Oxidized Structure	a(Å)	b(Å)	c(Å)
Cr_2_O_3_	4.944 ^a^	4.944	13.800
	4.955 ^b^ [26]	4.955	--
	4.951 ^c^ [27]	4.951	13.566
ZrO_2_	5.181 ^a^	5.255	5.355
	5.184 ^b^ [32]	5.274	5.358
	5.169 ^c^ [33]	5.232	5.341
Mn_3_O_4_	5.777 ^a^	5.777	9.525
	5.760 ^b^ [34]	5.774	9.580
	5.765 ^c^ [35]	5.765	9.442

^a^—This work; ^b^—results of first principles calculations; ^c^—experimental result.

## Data Availability

The original contributions presented in the study are included in the article, further inquiries can be directed to the corresponding authors.
